# 

**Published:** 1800-08

**Authors:** 


### NEW MEDICAL PUBLICATIONS IN JULY.

Obfervations on the Cow-pox, by William Woodville, M. D. Phyfician to the Small-pox and Inoculation Hofpitals. Phillips.

Reflections on the Cow-pox, illuftrated by Cafes to prove it an abfolute Security againft the Small-pox; in a Letter to Dr. Jenner from William Fermor, Efq. Robfon.

Nofology; or, a Syftematic Arrangement of Difeafes, by Claffes, Orders, Genera, and Species, with the diftinguifhing Characters of each, and Outlines of the Syftem of Sauvages, Linnæus, Vogel, Sagar, and Macbride; tranflated from the Latin of William Cullen, M. D. 6s. bds. Kay.

Refearches, Chemical and Philofophical, chiefly concerning Nitrous Oxyde, or dephlogifticated Nitrous Air, and its Refpiration; by Humphrey Davy, Superintendant of the Medical Pneumatic Inftitution, 8vo. 10s. 6d. boards. Johnfon.

The Doctrine of Phlogifton eftablifhed, and that of the Compofition of Water refuted; by Jofeph Prieftley, LL. D. 2s. 6d. Johnfon.

### NEW MEDICAL PUBLICATIONS IN GERMANY.

*Syftem der Chirurgie*, i. e. Syftem of Surgery; by J. ARNEMAN, Profeffor at Gottingen, Vol. I. with plates. Gottingen, 1799, 8vo. pp. 350. Vandenhock and Ruprecht.

*Verfuch einer Pragmatifchen Jefchichte*, i. e. Effay of a Pragmatical Hiftory of Medicine; by KURT SPRENGEL; fourth Volume, 1799, pp. 564, 8vo. price two rix-doll. or about 7s. Halle, for Gebauen.

*Bertrage zur Medicinischen Klinik*; i. e. Contributions to Clinick Medicine, collected on his Travels through Germany, Swifferland, and France, by Dr. ERNEST HORN, of Brunfwick, Vol. I. 1800, pp. 600, 8vo. price one rix-doll. twenty gr. or about 6s. Brunfwick, for Reichard.

*Elementarlehre der Organifchen Natur*; i. e. Elementary Syftem of Organic Nature; by Dr. FRANCIS JOSEPH SCHELVER, firft Volume, Organomie, 1800, pp 103, 8vo. price twelve gr. or about 2s. Gottingen, for Dieterich.

---

### To CORRESPONDENTS.

*We again requeft our Correfpondents to be particular in their manner of writing proper names of perfons and places, as feveral inaccuracies have arifen from a want of diftinctnefs in the character.*

*Communications have been received from Dr. Cappe, Dr. Gillespie, Dr. Sugrue, and Mr. Guthrie.*

